# Investigation of the Properties and Microstructure of EVA-Modified Underwater Repair Mortar

**DOI:** 10.3390/polym18151848

**Published:** 2026-07-28

**Authors:** Suining Zheng, Jiming Xiao, Huaxin Chen, Anhua Xu, Ruiyang Wang, Fulu Li

**Affiliations:** 1 College of Hydraulic and Civil Engineering, Qinghai Polytechnic University, Xining 810003, China; 2 Qinghai Provincial Key Laboratory of Tibet Plateau Highway Construction and Maintenance Technology, Xining 810003, China; 3 School of Materials Science and Engineering, Chang’an University, Xi’an 710061, China; 4 Qinghai Provincial Traffic Control Construction Engineering Group Co., Ltd., Xining 810021, China

**Keywords:** ethylene–vinyl acetate, underwater repair mortar, flexural bonding, microstructure, polymer modification

## Abstract

Ethylene–vinyl acetate (EVA) as a redispersible polymer can improve the workability, mechanical properties, and interfacial bonding of repair mortar, but the regulatory effect of different EVA dosages on underwater performance remains unclear. This study investigates EVA–polyacrylamide (PAM) composite-modified repair mortar and systematically evaluates the influence of varying EVA content on mortar performance. Multi-scale methods including setting time measurement, flowability test, flexural and compressive strength tests, underwater flexural bonding strength, water-to-air strength ratio, cement loss rate, porosity, and scanning electron microscopy (SEM) characterization were employed. The results indicate that mortar with 8% EVA exhibits the optimal overall performance. The initial setting time of the cement paste increased from 35 to 47 min, and the final setting time from 55 to 72 min, improving workability. In terms of mechanical properties, the 28-day flexural strength increased from 7.5 to 9.2 MPa, and the compressive strength from 44.4 to 50.6 MPa. Regarding underwater performance, the 28-day underwater flexural bonding strength rose from 5.1 to 6.5 MPa; the water-to-air strength ratio increased from 79.1% to 95.2%, and cement loss rate decreased from 3.18% to 0.32%; pH changes were maintained within a moderate range. Microstructural analysis shows that the total porosity at 28 d decreased from 16.8% to 13.5% for mortar containing 8% EVA, and SEM observations revealed that hydration products uniformly filled pores forming a dense network, consistent with the trends of mechanical and interfacial bonding performance enhancement.

## 1. Introduction

Underwater concrete structures are widely used in bridges, wharves, ports, tunnels, and various hydraulic engineering facilities, serving as critical materials to ensure the safety of water conservancy, transportation, and infrastructure [1]. Due to prolonged exposure to aquatic environments, these structures not only bear conventional loads but also face multiple environmental challenges, including fluid scouring, salt corrosion, freeze–thaw cycles, and temperature fluctuations [2,3]. During service, underwater concrete is prone to surface and internal cracks, voids, spalling, and local damage, which reduce structural mechanical performance and durability, and in severe cases may lead to structural failure or safety incidents. The formation and propagation of cracks not only weaken the load-bearing capacity but also provide pathways for water, chloride ions, and sulfate ions, accelerating steel reinforcement corrosion and material deterioration, thereby shortening the service life of the structures [4,5]. Therefore, effective repair and strengthening of underwater concrete structures, particularly early crack sealing and enhancement of interfacial properties, are of vital importance for ensuring long-term stability and extending service life.

Conventional underwater concrete repair primarily relies on cement-based mortars or cement pastes for filling and strengthening, as these methods offer ease of construction, relatively low cost, and good compatibility with the existing concrete substrate [6]. However, under underwater conditions, traditional mortars exhibit significant limitations during construction: the paste is easily washed out by flowing water, cement particles tend to segregate, interfacial bonding is insufficient, and early strength development is slow, resulting in repair performance that often fails to meet long-term durability requirements [7]. Moreover, the complex underwater environment, including fluid disturbances and prolonged immersion, significantly affects particle distribution within the paste. Consequently, conventional mortars demonstrate poor anti-dispersion properties and uneven interface bonding during the early hardening stage, compromising the overall compactness and structural stability of the repair layer [8]. These challenges not only limit repair efficiency but also increase engineering risks and maintenance costs. Therefore, there is an urgent need to develop high-performance repair mortars with excellent workability, underwater stability, and rapid early strength development to ensure the mechanical performance and durability of underwater structures during both early and long-term service stages.

To overcome the limitations of conventional cement-based repair materials in terms of workability, interfacial bonding, and durability, researchers have proposed various modification strategies, including mineral admixture optimization, fiber reinforcement, and polymer modification [9,10,11]. Among these, polymer modification has received considerable attention due to its ability to regulate the properties of the cement matrix at the microstructural level. Studies have shown that polymer emulsions can form continuous or semi-continuous polymer film structures within cement-based materials, interpenetrating with cement hydration products to create a spatial network with flexibility and toughness [12]. On one hand, the polymer films can fill capillary channels, reduce porosity, and increase material density, thereby enhancing impermeability and durability. On the other hand, the polymers themselves possess good deformability and interfacial adhesion, allowing them to buffer stress concentrations and suppress crack propagation under external loads, thus improving the flexural strength and toughness of the material [13,14]. Furthermore, the incorporation of polymers can improve paste flowability and particle dispersion, enhancing workability and reducing early paste washout [15]. Therefore, polymer-modified repair mortars have been widely applied in bridge repairs, waterproofing projects, and structural reinforcement, and are recognized as an important approach for improving the comprehensive performance of repair materials [16,17,18].

With the development of polymer modification technology, various polymer materials have been applied to repair mortar systems [19]. Among these, polyacrylamide (PAM), as a common anti-dispersant, can increase the paste viscosity and improve particle dispersion, thereby reducing paste washout and particle segregation during underwater construction, and enhancing workability stability and anti-dispersion performance [20]. Ethylene–vinyl acetate (EVA) exhibits excellent film-forming ability and flexibility, and can form a continuous polymer film structure during hardening, improving interfacial adhesion and optimizing pore structure, thus enhancing both the mechanical properties and microstructural continuity of the mortar [21,22,23]. Previous studies indicate that PAM effectively improves underwater workability stability, whereas EVA significantly enhances bonding performance, crack resistance, and durability of repair mortars [24,25]. However, most existing research focuses on single-polymer systems or above-water repair conditions. Systematic studies on PAM–EVA composite-modified underwater repair mortars remain scarce, especially regarding early-age hardening, cement washout, water–air strength ratio, and underwater interfacial bond performance. The microstructural evolution and synergistic mechanisms of this composite system have not been fully elucidated. Therefore, it is necessary to conduct systematic investigations on PAM–EVA composite-modified underwater repair mortars to reveal their regulation mechanisms on workability, underwater stability, interfacial bonding, and microstructural evolution.

In this study, a composite modification system using PAM and EVA was employed to systematically investigate the performance of underwater repair mortar. Under a fixed PAM dosage, different underwater repair mortar formulations were prepared by varying the EVA content. A comprehensive evaluation of workability, mechanical performance, underwater stability, and interfacial bonding was conducted, including tests on setting time, flowability, flexural and compressive strength, cement loss rate, solution pH, water–air strength ratio, and underwater flexural bonding strength. Additionally, the microstructural characteristics of the mortar were analyzed using porosity measurements and scanning electron microscopy (SEM) to reveal the regulation of pore continuity, hydration product distribution, and interfacial density by EVA. By integrating macroscopic performance and microstructural analysis, the study further elucidates the mechanisms and synergistic effects of PAM–EVA composite modification in underwater repair mortars.

## 2. Materials and Methods

### 2.1. Materials

The sulfoaluminate cement used in this study was SAC 42.5 and the ordinary Portland cement was P·O 42.5; both were purchased from Hubei Huaxin Cement Co., Ltd (Wuhan, China). Natural river sand with a fineness modulus of approximately 2.5 was used as the fine aggregate after being washed and air-dried. Tap water was used for mixing. PAM, a high-molecular-weight water-soluble polymer, served as an anti-dispersant and was supplied by Shanghai Qichen Chemical Technology Co., Ltd (Shanghai China). A polycarboxylate-based high-performance water reducer in liquid form with a solid content of 8% was provided by Wuhan Sanyuan Special Building Materials Co., Ltd (Wuhan, China). EVA re-dispersible powder, purchased from Shanghai Qichen Chemical Technology Co., Ltd. (Wuhan, China), was used as the polymer-modified material. The chemical compositions of the two cements are summarized in Table 1.

### 2.2. Specimen Preparation

Specimens of underwater repair mortar were prepared according to the designed mix proportions. First, the required amounts of cement, sand, PAM, water reducer, and water were weighed according to the mix design. Cement, sand, and PAM were thoroughly mixed before adding water and the water reducer, and the mixture was stirred until a homogeneous mortar was formed. EVA re-dispersible powder was then incorporated, and mixing continued to ensure complete and uniform dispersion. The prepared mortar was poured into standard molds measuring 160 mm × 40 mm × 40 mm, and lightly tapped to eliminate air bubbles and ensure full mold compaction. After casting, the specimens were left to stand for approximately one hour until the surface had partially hardened, then demolded and immediately transferred to an underwater environment for curing. All specimens were immersed in water at 20 ± 2 °C for a curing period of 28 d.

To evaluate the influence of EVA content on the performance of the repair mortar, four different EVA dosages were tested, namely 0%, 4%, 8%, and 12%, the mix proportions were shown in Table 2. Each dosage group included three replicate specimens to ensure data reliability and experimental reproducibility. During the curing period, the specimens remained fully submerged, and water levels and quality were checked periodically to maintain stable curing conditions.

### 2.3. Setting Time Test

Setting time is an important indicator for evaluating the hardening rate of underwater repair mortars. In this study, cement paste was used for the determination of setting time to avoid interference from sand. The mix ratios of the cement paste specimens were consistent with the above-described mortar formulations. The water-to-cement ratio was 0.45, and 2% PAM was selected, while the EVA content was set at 0%, 4%, 8%, and 12%.

The setting time was measured using a Vicat apparatus, with the needle method employed to determine the initial and final setting times of the cement paste. Cement paste specimens were prepared according to the mix ratios and thoroughly mixed, then poured into standard Vicat molds. After initial stirring, the needle was periodically brought into contact with the surface of the paste to observe its penetration depth until it could no longer penetrate. The initial and final setting times of each paste specimen were recorded to analyze the influence of different EVA contents on the setting behavior of the repair mortar. This test allows the evaluation of the workability of underwater repair mortar during construction and provides theoretical support for subsequent performance analysis.

### 2.4. Flowability Test

According to the “Test Standard for Polymer-Modified Cement Mortar” (DL/T 5126-2001), the flowability of the repair mortar was evaluated by measuring the spread diameter [26]. Underwater repair mortar specimens were prepared according to the designed mix ratios and poured into standard circular molds. Subsequently, a standard jumping table method was applied for a specified number of jolts, allowing the mortar to uniformly spread within the mold. The maximum spread diameter was then recorded. By testing the flowability of repair mortar with different EVA contents, the workability and fluidity under underwater conditions were assessed, ensuring that the repair mortar could effectively fill cracks and facilitate construction.

### 2.5. Flexural and Compressive Strength Tests

To evaluate the mechanical performance of the repair mortar, flexural and compressive strength tests were conducted. All specimens measured 40 mm × 40 mm × 160 mm and were prepared according to the designed mix ratios. The flexural strength was determined using a three-point bending method, where a uniform bending load was applied to the specimen until failure occurred, and the maximum flexural stress was recorded. After the flexural test, the two fractured halves of each prism were used for the compressive strength test in accordance with the standard procedure for cement mortar testing. Since the compressive load was applied to the fractured halves rather than the original prism, the prior flexural loading did not significantly affect the measured compressive strength. A uniform compressive load was applied until the specimen failed, and the maximum compressive stress was recorded. Flexural and compressive strengths were measured for specimens cured for 1, 3, 7, 14, and 28 d to investigate the evolution of mechanical performance over time.

### 2.6. Underwater Anti-Dispersibility Tests

Underwater anti-dispersibility is a key indicator for evaluating the stability of repair mortar in submerged environments. In this study, the underwater anti-dispersibility of repair mortar was assessed through the water–air strength ratio, cement loss rate, and solution pH.

#### 2.6.1. Cement Loss Rate Test

**Figure 1:**
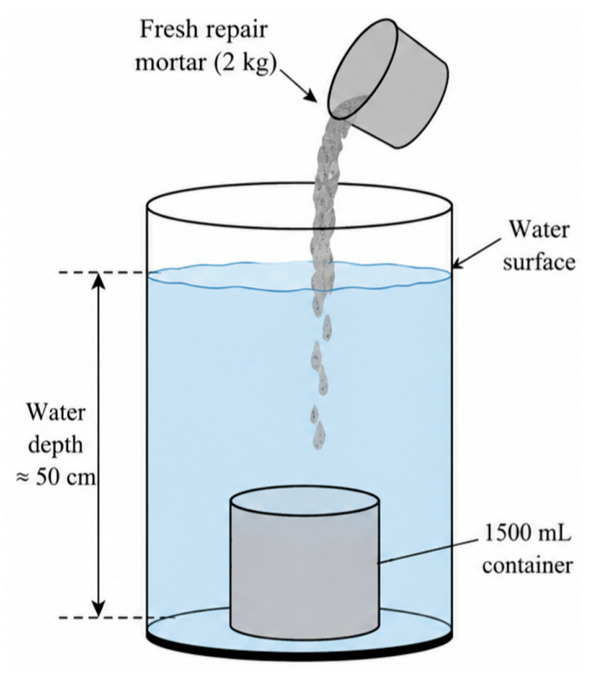
Schematic diagram of the cement loss test setup.

The cement washout test evaluates the stability of repair mortar in water. Approximately 50 cm of water was poured into a water tank, with a 1500 mL container placed at the bottom. Then, 2 kg of freshly mixed underwater repair mortar was poured from the water surface into the container, ensuring that the mortar fully entered the container without spillage. After standing for 5 min, the container was removed, residual water was drained, and the combined mass of the container and mortar was weighed [27]. A Schematic diagram of the cement loss test setup is shown in Figure 1. Cement loss rate was calculated according to Equation (1):

(1) $$∆\beta =\frac{m_{1}-m_{2}}{m_{1}-m_{3}}$$

where m_1_ is the mass of the container and mortar before immersion (g), m_2_ is the mass of the container and mortar after immersion (g), m_3_ is the mass of the container (g), and ∆β represents the cement loss rate (%). Lower washout values indicate better underwater stability of the repair mortar.

#### 2.6.2. Solution pH Measurement

The change in solution pH is an important indicator to assess the stability and anti-dispersibility of underwater repair mortar. During cement hydration, alkaline components dissolve into the surrounding water, affecting its pH. The pH test was conducted by pouring 800 mL of deionized water into a 1000 mL beaker. Then, 500 g of freshly mixed repair mortar was divided into 10 equal portions and quickly poured into the water to ensure uniform dispersion of the cement paste. After standing for 3 min, 600 mL of the solution was extracted using a pipette, and the pH was measured with a pH meter.

#### 2.6.3. Water–Air Strength Ratio

The water–air strength ratio test characterizes the anti-dispersibility of cement mortar under water relative to air. Specimens were prepared using submerged casting and air casting methods, and compressive strength tests were conducted at the same curing ages. The water–air strength ratio was calculated as the ratio of underwater compressive strength to that of air-cured specimens at 1 d, 3 d, and 28 d. Higher values indicate better underwater anti-dispersibility.

### 2.7. Underwater Bonding Performance Test

**Figure 2:**
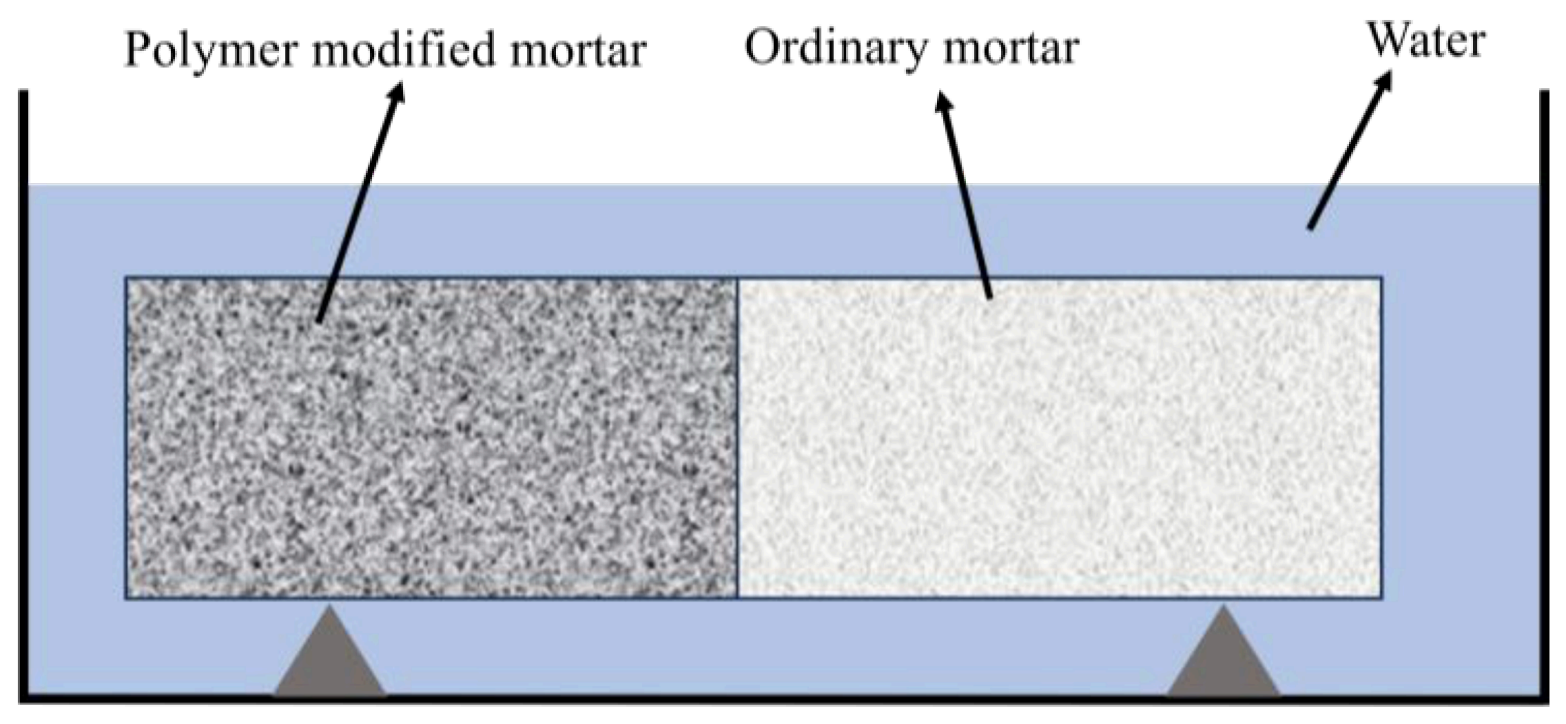
Schematic of underwater bonding between new and existing mortar.

The bonding performance of underwater repair mortar is a key indicator for evaluating repair effectiveness. In this study, the underwater flexural bonding strength test was used to assess the adhesion between the repair mortar and substrate at different curing ages. First, ordinary Portland cement mortar specimens were prepared and subjected to a 7 d underwater curing period. After the flexural strength test, half of the specimen cross-section was submerged in water and returned to the mold to maintain a moist interface. The modified repair mortar mixture was then cast into the mold to bond with the substrate and immediately placed underwater for curing. A schematic of the bonded specimens is shown in Figure 2. The molds were removed after 1 d of curing, and the specimens were continuously submerged in water. Underwater flexural strength tests were conducted at 1 d, 3 d, 7 d, 14 d, and 28 d. The measured values at each curing age directly represent the underwater flexural bonding strength, which allows evaluation of the adhesion performance of repair mortar with different EVA contents.

### 2.8. Porosity Test

The porosity test was conducted to evaluate the pore structure and compactness of the underwater repair mortar, providing insight into its impermeability and durability. The water-saturation method was employed to measure the porosity of mortars at different curing ages. Specimens were prepared and taken out after curing for 1 d, 3 d, and 28 d. Each specimen was dried to a constant weight and then immersed in water to ensure full saturation. After a certain period, the specimens were removed, surface water was wiped off, and the saturated mass was recorded. Subsequently, the dry mass was measured [28]. Porosity was calculated using the following Equation (2):

(2) $$P=(\frac{M_{0}-M_{i}}{V}\times \rho_{w})\times 100\%$$

where M_0_ is the mass of the fully saturated specimen (g), M_i_ is the mass of the dried specimen (g), V is the specimen volume (cm^3^), $\rho_{w}$ is the water density (g/cm^3^), and P is the porosity (%).

### 2.9. Microstructural Analysis

To investigate the microstructure of the underwater repair mortar, SEM was employed on mortars after 28 d of curing. Small specimens were cut from each repair mortar with different EVA contents. The specimens were dried to constant weight and then coated with a conductive metal layer to ensure proper conductivity. The prepared specimens were subsequently scanned using SEM to observe the microstructural morphology of the repair mortar.

## 3. Results and Discussion

### 3.1. Setting Time of Cement Paste

**Figure 3:**
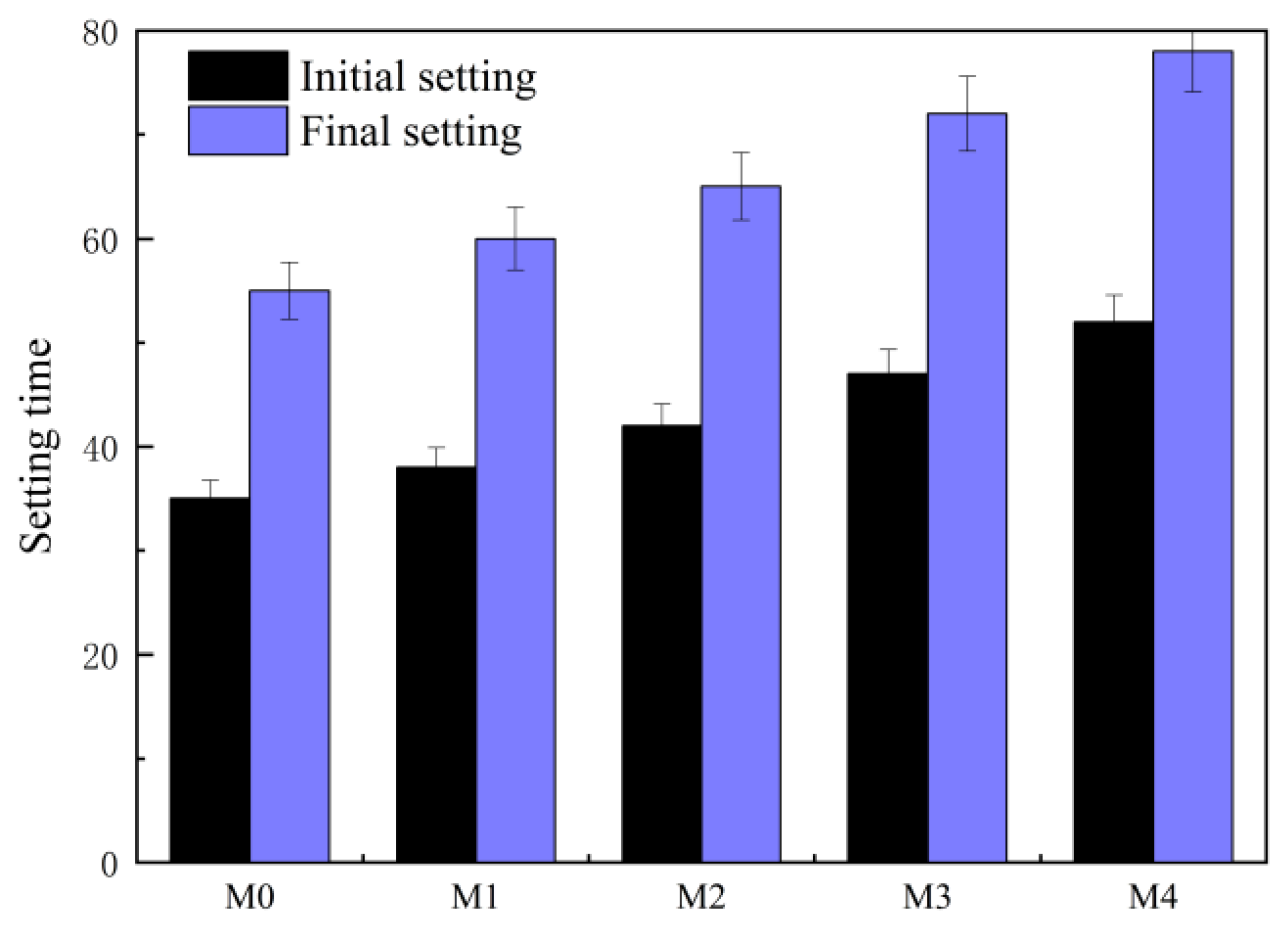
Setting times of polymer-modified cement paste.

Figure 3 illustrates the influence of varying EVA contents on the setting time of the repair mortar. As the EVA content increased, both the initial and final setting times of the cement paste gradually extended, indicating that EVA significantly delayed the cement hydration process. For M0, the initial setting time was 35 min and the final setting time was 55 min. After adding 2% PAM, the initial and final setting times of M1 were 38 min and 60 min, respectively, showing a slight extension. PAM primarily improves the workability of the cement paste by enhancing particle dispersion and reducing agglomeration, but its effect on the hydration rate is limited because its main function is dispersing rather than significantly altering the hydration kinetics. For M2, with EVA addition, the initial and final setting times increased to 42 min and 65 min, demonstrating a more pronounced delay. EVA, as a polymer modifier, forms a film-like structure within the cement paste, reducing the contact area between cement particles and water, thereby slowing down the hydration reaction and extending the setting time. This effect is achieved by altering the paste’s cohesion and particle dispersion, making it more difficult for cement particles to fully react with water, thus lowering the hydration rate [29]. In M3, the initial and final setting times were 47 min and 72 min, respectively, showing a further extension trend. This suggests that with increasing EVA content, the cement paste structure becomes denser, further delaying hydration. For M4, the initial and final setting times were 52 min and 78 min, respectively, exhibiting a similar prolongation. This may be attributed to the excessive EVA forming a more compact polymer layer in the paste, which further restricts the contact between cement particles and water, thereby significantly inhibiting the hydration reaction and prolonging the setting time.

### 3.2. Flowability of Repair Mortar

**Figure 4:**
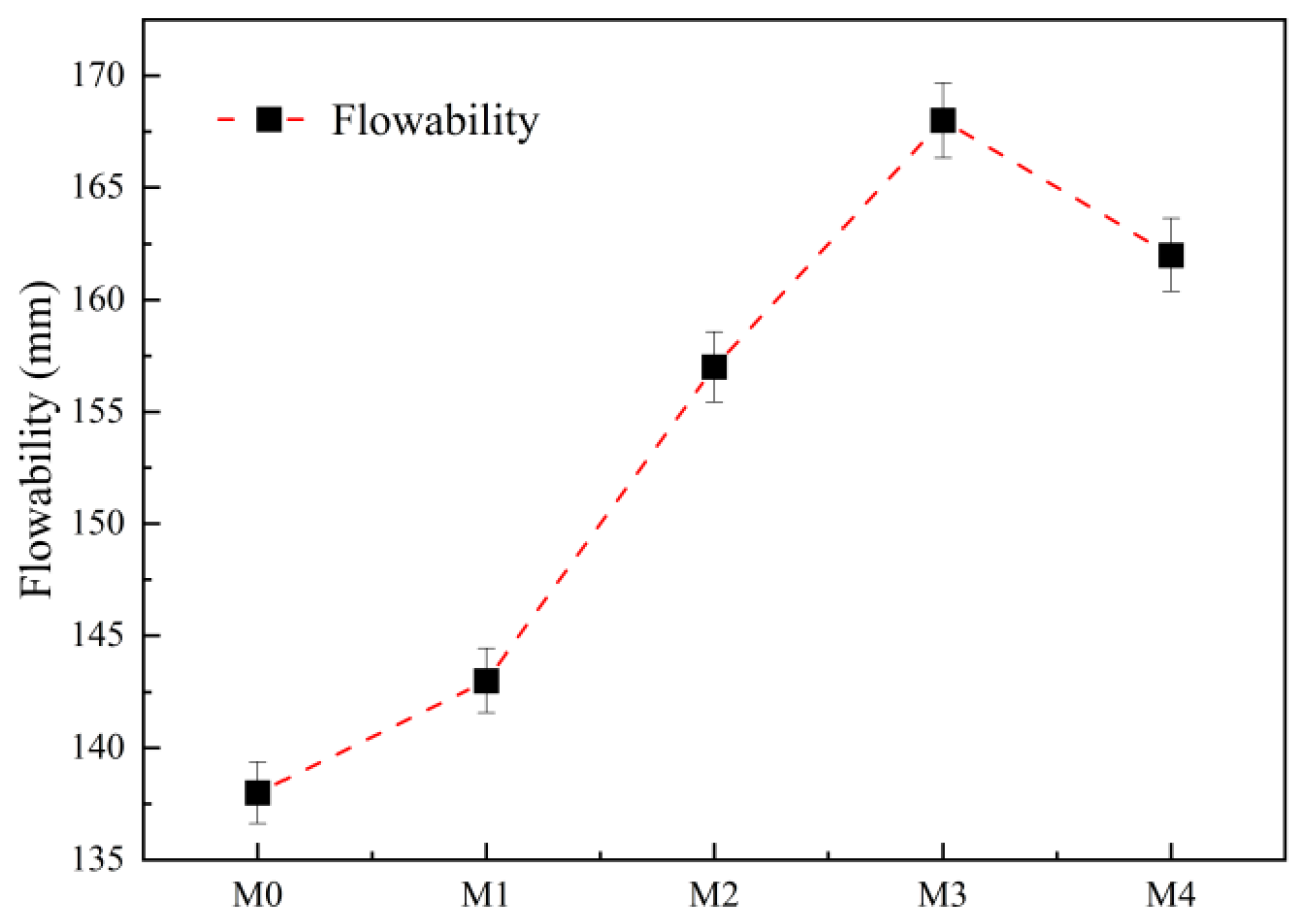
Flowability of polymer-modified repair mortar.

Figure 4 shows the effect of different EVA contents on the flowability of the repair mortar. With increasing EVA dosage, the flow spread of the mortar increased significantly. M0 had a flow spread of 138 mm, reflecting the typical flow behavior of ordinary cement mortar. M1 had a flow spread of 143 mm, indicating that PAM moderately improved the dispersion of cement particles. The role of PAM mainly lies in enhancing particle dispersion and reducing agglomeration, which slightly increases flowability. M2 had a flow spread of 157 mm, showing a notable improvement, as EVA reduces particle agglomeration and promotes better contact between cement particles and water. M3 had a flow spread of 168 mm, indicating that an appropriate amount of EVA can effectively enhance the flowability of the cement paste and improve the workability of the repair mortar. M4 had a flow spread of 162 mm. Although still higher than M0, the reduced increase suggests that excessive EVA may form a dense polymer film in the cement paste, limiting cement hydration and further reducing flow spread. Controlling the EVA dosage within an appropriate range is critical for optimizing the flowability of repair mortar. Moderate EVA content significantly improves flow spread, enhancing workability and penetration ability, whereas excessive EVA may reduce the marginal improvement and inhibit hydration, ultimately affecting repair performance.

### 3.3. Mechanical Performance of Repair Mortar

#### 3.3.1. Flexural Strength

**Figure 5:**
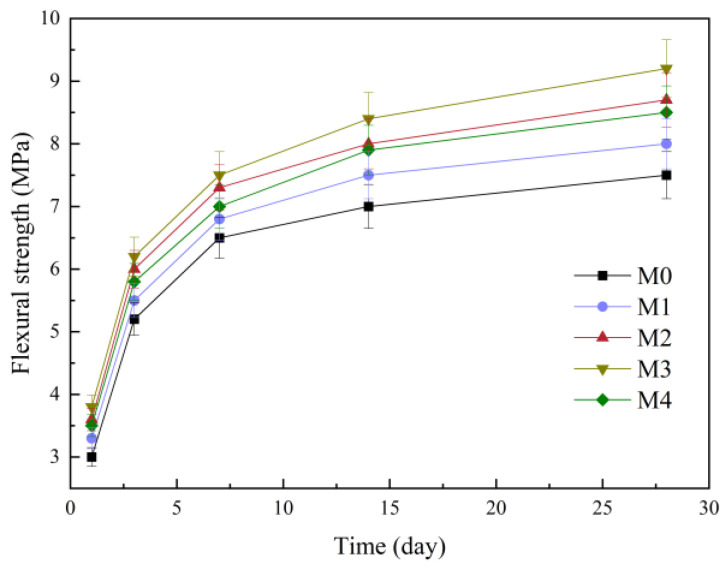
Flexural strength of polymer-modified repair mortar.

Figure 5 presents the evolution of flexural strength of the polymer-modified repair mortar over different curing periods. With increasing EVA dosage, the flexural strength of the repair mortar gradually improved. Specifically, M0 exhibited flexural strengths of 2.8 MPa at 1 d and 3.0 MPa at 3 d, gradually increasing with curing time to reach 7.5 MPa at 28 d. M1 showed flexural strengths of 3.0 MPa at 1 d and 3.3 MPa at 3 d, reaching 8.0 MPa at 28 d, indicating that the incorporation of PAM slightly enhanced early flexural performance. Compared with M0, PAM improved the dispersion of cement particles during hydration, but its contribution to flexural strength enhancement was limited. For M2, the flexural strengths were 3.2 MPa at 1 d and 3.6 MPa at 3 d, reaching 8.7 MPa at 28 d, showing a notable improvement. With increasing EVA content, its effect became more pronounced, as EVA enhanced particle dispersion, reduced agglomeration, and promoted contact between cement particles and water, thereby accelerating hydration and increasing flexural strength [30]. M3 exhibited flexural strengths of 3.5 MPa at 1 d and 4.0 MPa at 3 d, reaching 9.2 MPa at 28 d, further confirming that moderate EVA addition significantly improves the flexural strength of repair mortar. For M4, the flexural strengths were 3.1 MPa at 1 d and 3.5 MPa at 3 d, reaching 8.5 MPa at 28 d. Although the strength was still higher than M0, the increment decreased, and the 28 d value was slightly lower than M3, suggesting that excessive EVA may form a dense polymer film in the cement paste, limiting the contact between particles and water and inhibiting hydration, thus restricting further flexural strength enhancement.

#### 3.3.2. Compressive Strength

**Figure 6:**
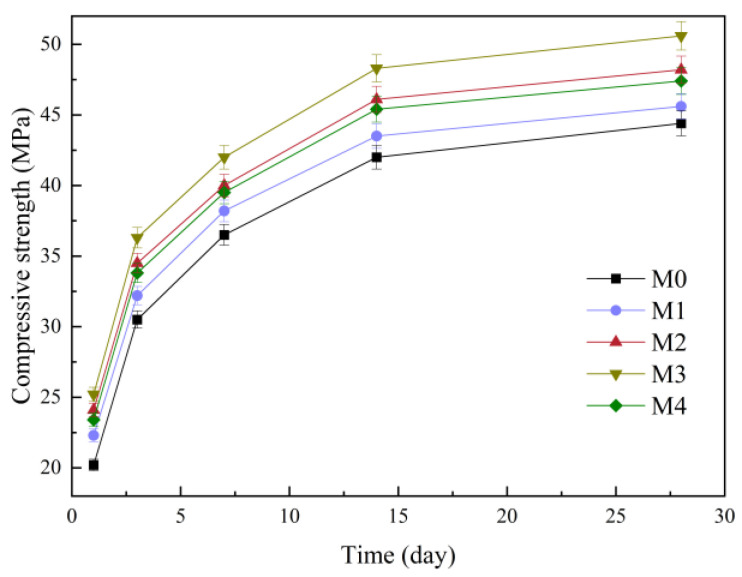
Compressive strength of polymer-modified repair mortar.

Figure 6 shows the compressive strength of polymer-modified repair mortar with different EVA contents over the curing period. At 1 d, the compressive strength of M0, M1, M2, M3, and M4 was 20.2 MPa, 22.3 MPa, 24.1 MPa, 26.4 MPa, and 25.0 MPa, respectively. Compared with M0, the addition of EVA significantly increased the compressive strength, especially for M3, indicating that EVA enhanced the bonding between cement particles, and improved particle dispersion, thus enhancing compressive performance. At 3 d, the compressive strength of M0, M1, M2, M3, and M4 was 22.5 MPa, 24.1 MPa, 26.5 MPa, 28.2 MPa, and 26.6 MPa, respectively. At this stage, M3 still showed the highest increase, demonstrating that an appropriate EVA content can further improve compressive strength. For M4, although the strength continued to increase, the increment was reduced, which may be due to the formation of a denser polymer film that limits cement particle contact with water and inhibits hydration, thus restricting further compressive strength enhancement. At 28 d, the compressive strength of M0, M1, M2, M3, and M4 was 44.4 MPa, 45.6 MPa, 48.2 MPa, 50.6 MPa, and 47.4 MPa, respectively. M3 exhibited the highest compressive strength, indicating that an appropriate EVA content not only optimized the hydration rate but also further improved cement hardening at later stages. The strength increment for M4 was significantly reduced, indicating that excessive EVA limited cement particle contact with water and hydration, which affected late-age strength development.

### 3.4. Underwater Dispersion Resistance of Repair Mortar

#### 3.4.1. Cement Loss Rate

**Figure 7:**
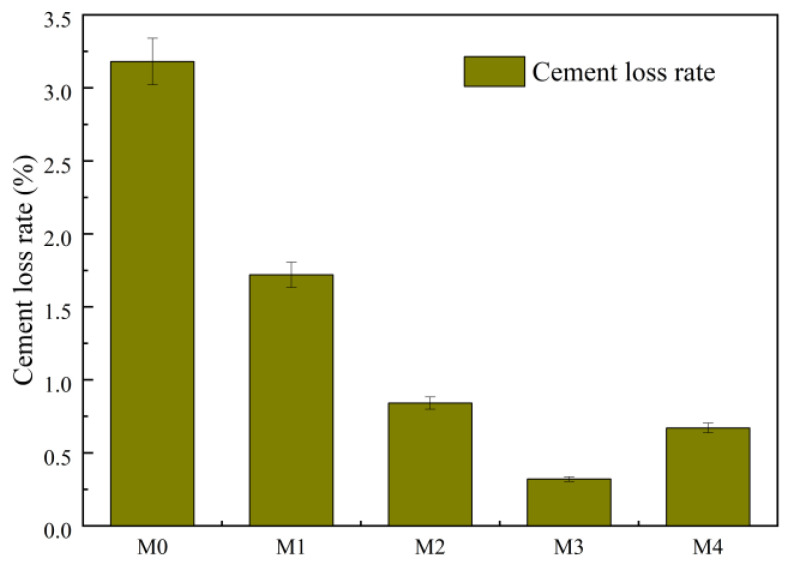
Cement loss rate of polymer-modified repair mortars under water.

Figure 7 shows the cement loss rate of repair mortars with different mix proportions. M0 exhibited the highest cement loss rate of 3.18%, indicating that the mortar without polymer was prone to particle washout under water, resulting in poor underwater stability. With the addition of PAM, the cement loss rate of M1 decreased to 1.72%, demonstrating that PAM effectively improved the cohesion of the mortar, reducing particle segregation and loss in water. This is primarily attributed to the formation of a spatial network structure by PAM, which enhances the encapsulation ability and resistance to washout of the mortar. With further addition of EVA, M2 and M3 showed further reductions in cement loss rate, reaching 0.84% and 0.32%, respectively, with M3 achieving the lowest value. This indicates that an appropriate amount of EVA can further enhance the underwater dispersion resistance of the repair mortar. EVA provides good dispersion and film-forming properties, gradually forming a polymer film during hydration that wraps and bridges cement particles, thus improving the overall stability of the mortar and reducing particle loss. However, when the EVA content was further increased to M4, the cement loss rate rose to 0.67%. This phenomenon suggests that excessive EVA may reduce the uniformity of the mortar’s internal structure and cause local polymer enrichment, weakening the cohesive structure and thus diminishing resistance to water-induced washout. Overall, the synergistic effect of PAM and EVA significantly reduces the cement loss rate of repair mortars, enhancing their underwater stability, with M3 demonstrating the optimal anti-dispersion performance.

#### 3.4.2. Solution pH

**Figure 8:**
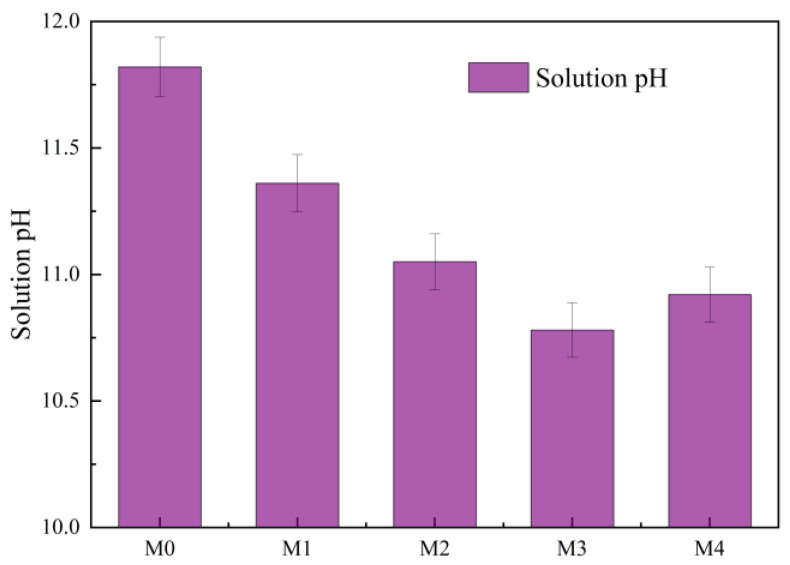
pH of polymer-modified repair mortar solution.

Figure 8 shows the pH variation of the solutions corresponding to different mix proportions of repair mortars. The pH of the solution for M0 was the highest at 11.82, indicating that the mortar without polymer was prone to leaching cement particles and alkaline ions into water, resulting in a significant increase in alkalinity. With the addition of PAM, the pH of M1 decreased to 11.36, demonstrating that PAM effectively improved the stability of the paste, reducing the dispersion and loss of cement particles and thus lowering the release of OH- and other alkaline ions. As EVA content increased, the pH values of M2 and M3 further decreased to 11.05 and 10.78, respectively, with M3 showing the lowest value. This indicates that an appropriate EVA dosage further enhanced the underwater anti-dispersive ability of the paste, minimizing direct contact between cement particles and the surrounding water and inhibiting alkaline ion leaching. In addition, the polymer film formed by EVA improved the continuity and density of the internal structure, hindering particle migration and ion diffusion, resulting in a continuous decrease in solution pH. When the EVA content increased to M4, the solution pH slightly rose to 10.92, suggesting that excessive EVA may lead to uneven polymer distribution within the paste, reducing the system’s stability and making some particles and ions more prone to migrate into the surrounding water. Overall, the variation in solution pH was consistent with the cement loss rate, indicating that both parameters effectively reflect the underwater anti-dispersive performance of the repair mortars. This correlation arises because both are governed by the degree of particle dispersion and ion leaching during underwater mixing; higher cement loss promotes the release of alkaline species such as OH^−^ into the surrounding water, leading to increased pH values. Therefore, the consistent trends between these two indicators further confirm the improved underwater stability provided by PAM–EVA composite modification.

#### 3.4.3. Water–Air Strength Ratio of Repair Mortars

**Figure 9:**
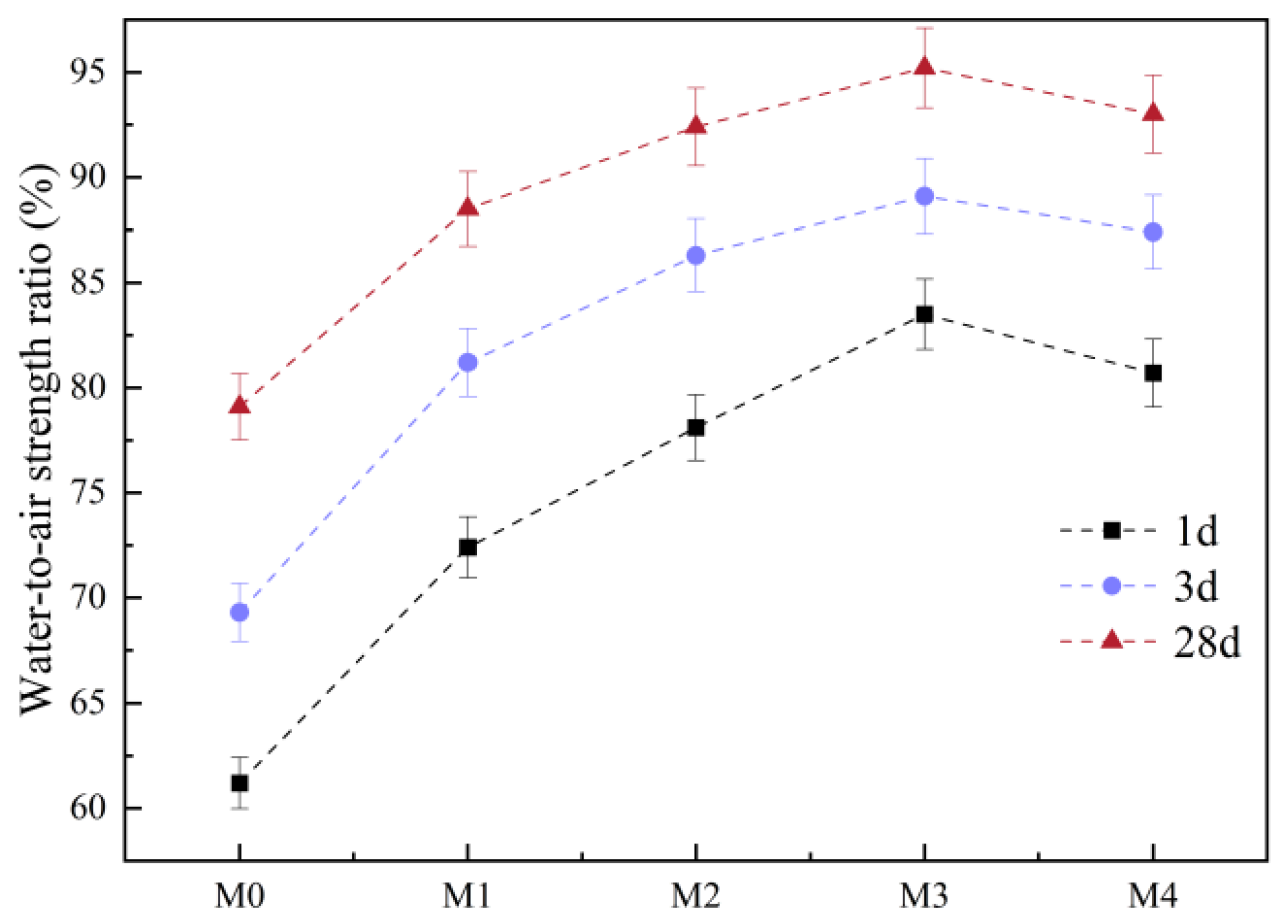
Water–air strength ratio of polymer-modified repair mortars.

Figure 9 shows the water–air strength ratio of repair mortars with different EVA contents at various ages. The results indicate that the water–air strength ratio of all mortar groups gradually increased with age, reflecting the progressive improvement in the internal structure under underwater curing and the approach of compressive strength to the level of air-cured specimens. At 1 d, the water–air strength ratio of M0 was only 61.2%, indicating that the unmodified mortar was prone to cement washout and structural defects in underwater conditions, resulting in significant early strength loss. With the addition of PAM, the ratio of M1 increased to 72.4%, suggesting that PAM effectively enhanced underwater stability, reduced particle segregation and cement loss, and improved early underwater strength. Further incorporation of EVA led to water–air strength ratios of 78.1% and 83.5% for M2 and M3, respectively, demonstrating a more pronounced improvement. This indicates that an appropriate EVA content can further optimize the mortar structure, enhance the bonding between cement particles, and increase the overall matrix density through film formation, thereby improving strength development in underwater conditions. At 3 d, the water–air strength ratios further increased, reaching 69.3%, 81.2%, 86.3%, 89.1%, and 87.4% for M0, M1, M2, M3, and M4, respectively. Compared with 1 d, the improvement was more pronounced for polymer-modified mortars, indicating that ongoing hydration allowed the polymer to gradually enhance interfacial bonding and structural stability, mitigating internal defects under underwater curing. M3 still exhibited the highest water–air strength ratio, showing the most effective structural optimization at this EVA content. At 28 d, the water–air strength ratios of all groups approached stabilization. M0 reached 79.1%, while M1, M2, M3, and M4 reached 88.5%, 92.4%, 95.2%, and 93.0%, respectively. M3 achieved the highest ratio, indicating that the optimal EVA content significantly enhanced underwater hardening and the integrity of the matrix at later ages. When the EVA content was further increased to M4, the water–air strength ratio slightly decreased compared with M3, suggesting that excessive EVA may form excess polymer phases within the matrix, interfering with effective bonding among hydration products and slightly limiting late-age strength development.

### 3.5. Underwater Bonding Performance of Repair Mortars

**Figure 10:**
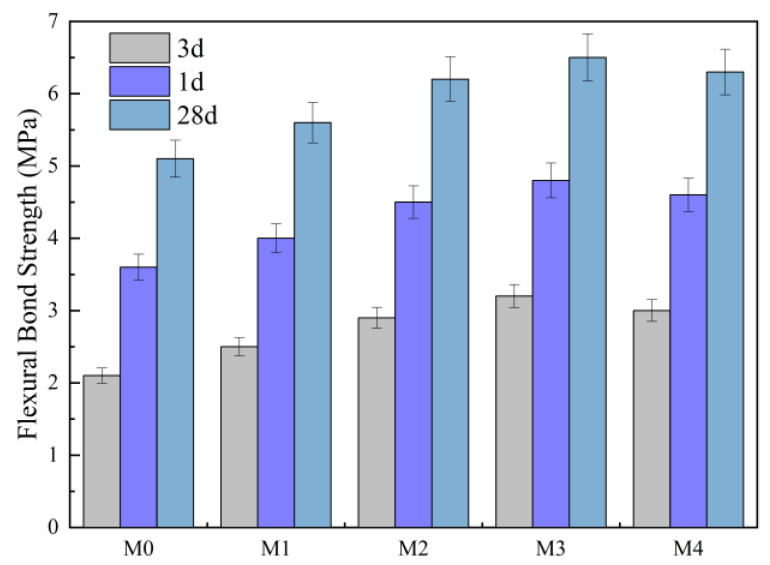
Flexural bonding strength of polymer-modified repair mortars under water.

Figure 10 shows the variation in flexural bonding strength at the interface between the repair mortars and the substrate under water with different EVA content over curing time. The flexural bonding strength of all mortar groups gradually increased with curing time, with faster growth in the early stage. At 1 d, the flexural bonding strength of M0, M1, M2, M3, and M4 was 2.1 MPa, 2.5 MPa, 2.9 MPa, 3.2 MPa, and 3.0 MPa, respectively. Early differences were evident, with M3 being the highest and M0 the lowest. At this stage, interfacial hydration was not fully developed. PAM improved initial bonding by enhancing mortar flowability and particle dispersion, while EVA in M2 and M3 began to promote particle contact and mechanical interlocking at the interface. At 3 d, the flexural bonding strength of M0, M1, M2, M3, and M4 reached 3.6 MPa, 4.0 MPa, 4.5 MPa, 4.8 MPa, and 4.6 MPa, respectively. With accelerated interfacial hydration, the polymer formed a continuous film at the interface, significantly improving cement particle dispersion and interfacial density. M2 and M3 exhibited the most notable increases, indicating that EVA promoted early interfacial hydration, pore filling, and microcrack reduction, thereby enhancing underwater interfacial bonding. M4 showed slight early improvement but was slightly lower than M3, suggesting that excessive EVA may accumulate at the interface, forming a local dense film that limits cement–water contact and reduces the early bonding increment. At 28 d, the flexural bonding strength of M0, M1, M2, M3, and M4 was 5.1 MPa, 5.6 MPa, 6.2 MPa, 6.5 MPa, and 6.3 MPa, respectively, with growth rates significantly slowing. Interfacial hydration approached saturation, and additional hydration products contributed minimally to bonding strength. M3 remained the highest, while M4 was slightly lower but still above M0 and M1. These results indicate that the polymer film formed at the interface under the optimal EVA content effectively improved interfacial density and structural integrity, enhancing underwater bonding performance. PAM further assisted by improving particle dispersion and mortar flowability, contributing to early interfacial bonding. The distinct early rapid increase followed by slower growth clearly illustrates the mechanism by which polymers reinforce the underwater repair mortar interface.

### 3.6. Porosity of Repair Mortars

**Figure 11:**
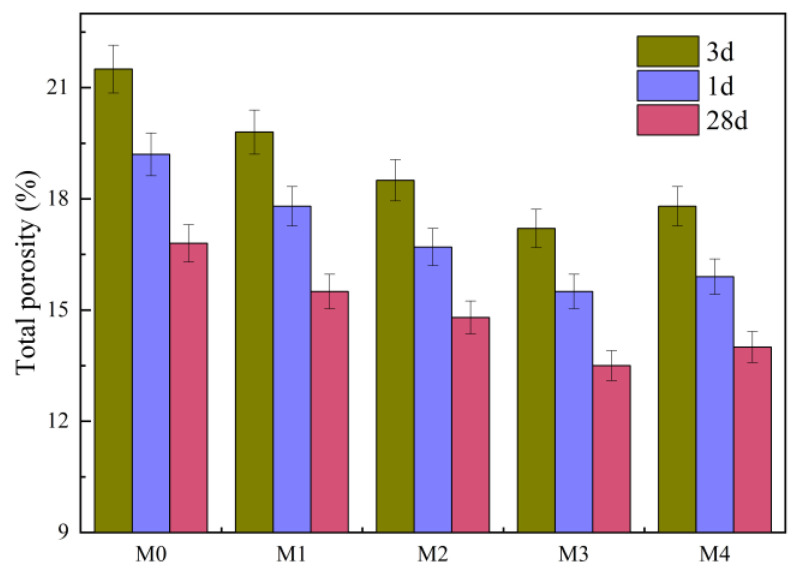
Total porosity of polymer-modified repair mortars.

Figure 11 shows the total porosity of repair mortars with different EVA contents over curing time. At 1 d, the porosity of M0 was 21.5%, significantly higher than that of the other mortars, due to particle aggregation and a loose interfacial structure in the unmodified mortar, resulting in more early-age pores. With the addition of 2% PAM, the porosity of M1 decreased to 19.8%, indicating that PAM improved cement paste dispersion and reduced particle aggregation, partially filling initial micropores. As EVA was incorporated, the porosity of M2 and M3 further decreased to 18.5% and 17.2%, respectively, demonstrating that the polymer formed a continuous film in the paste, enhancing particle contact and increasing interfacial density, thereby reducing pore volume. The porosity of M4 slightly increased to 17.8%, possibly due to local EVA aggregation forming thick films, resulting in uneven pore distribution and incomplete micropore filling. At 3 d, the porosity of M0 decreased to 19.2%, while M1, M2, M3, and M4 were 17.8%, 16.7%, 15.5%, and 15.9%, respectively, showing a noticeable decline. At this stage, hydration accelerated, and the polymer began forming dense interfacial films, improving paste structure and interfacial compactness, further reducing the porosity of underwater repair mortars. The porosity decrease was more pronounced in M2 and M3, indicating that optimal EVA content promoted sufficient cement particle contact and optimized the internal microstructure, whereas the slightly higher porosity in M4 reflected potential microstructural imbalance caused by excessive polymer. At 28 d, the porosity of M0, M1, M2, M3, and M4 was 16.8%, 15.5%, 14.8%, 13.5%, and 14.0%, respectively, reaching a relatively stable state. As curing progressed, interfacial hydration approached saturation, and additional hydration products contributed minimally to pore filling. M3 maintained the lowest porosity, showing that optimal EVA effectively improved the microstructure and interfacial density, enhancing the overall compactness and underwater durability of the mortars. The slightly higher porosity of M4 further confirmed that excessive EVA may cause local polymer aggregation, affecting cement particle contact and uniform hydration, slightly increasing porosity. The porosity trends correspond well with mechanical properties, interfacial bonding, and underwater stability, indicating that PAM and EVA modification directly improved the overall performance of the mortars.

### 3.7. Microstructure of Repair Mortars

**Figure 12:**
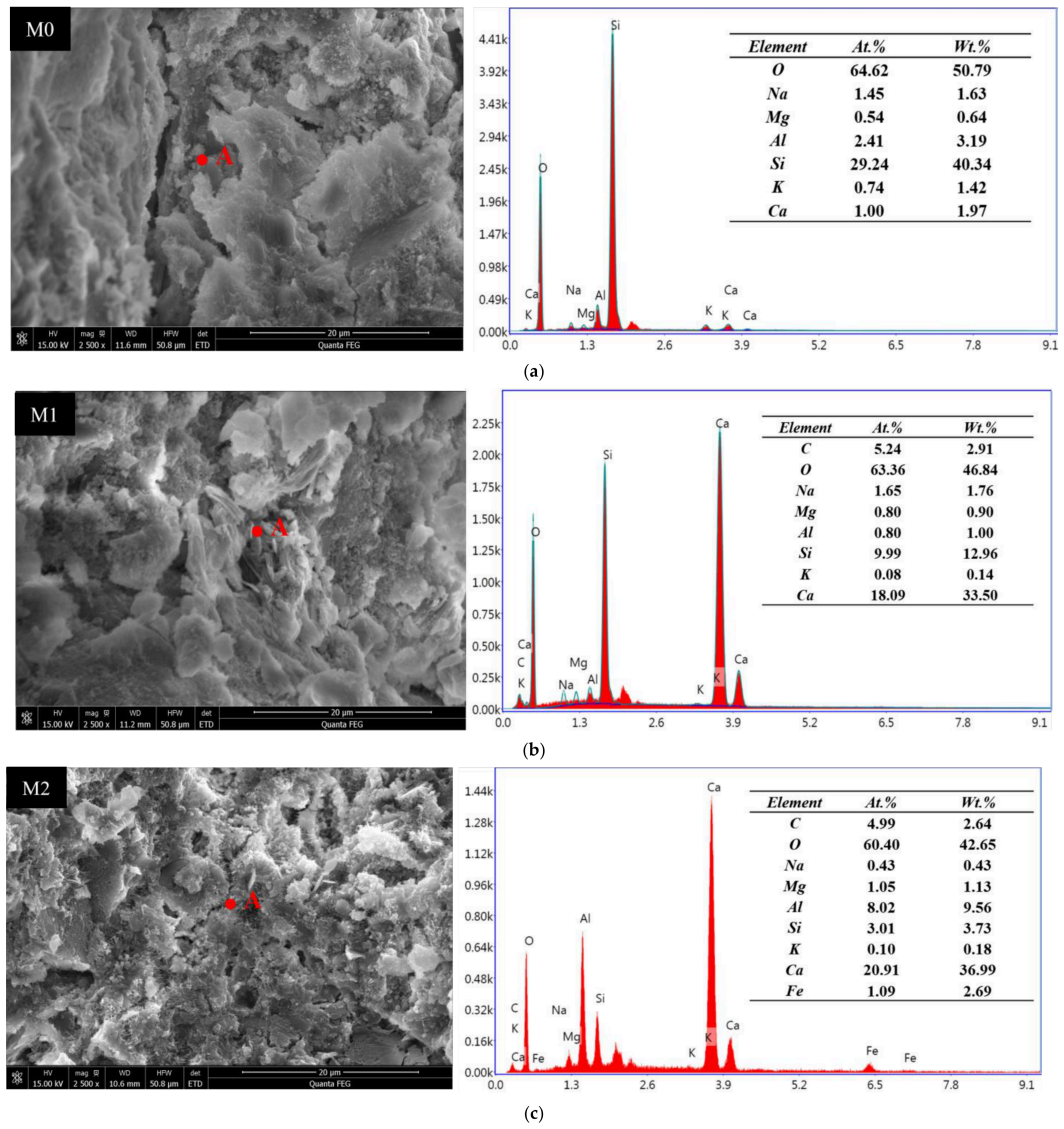

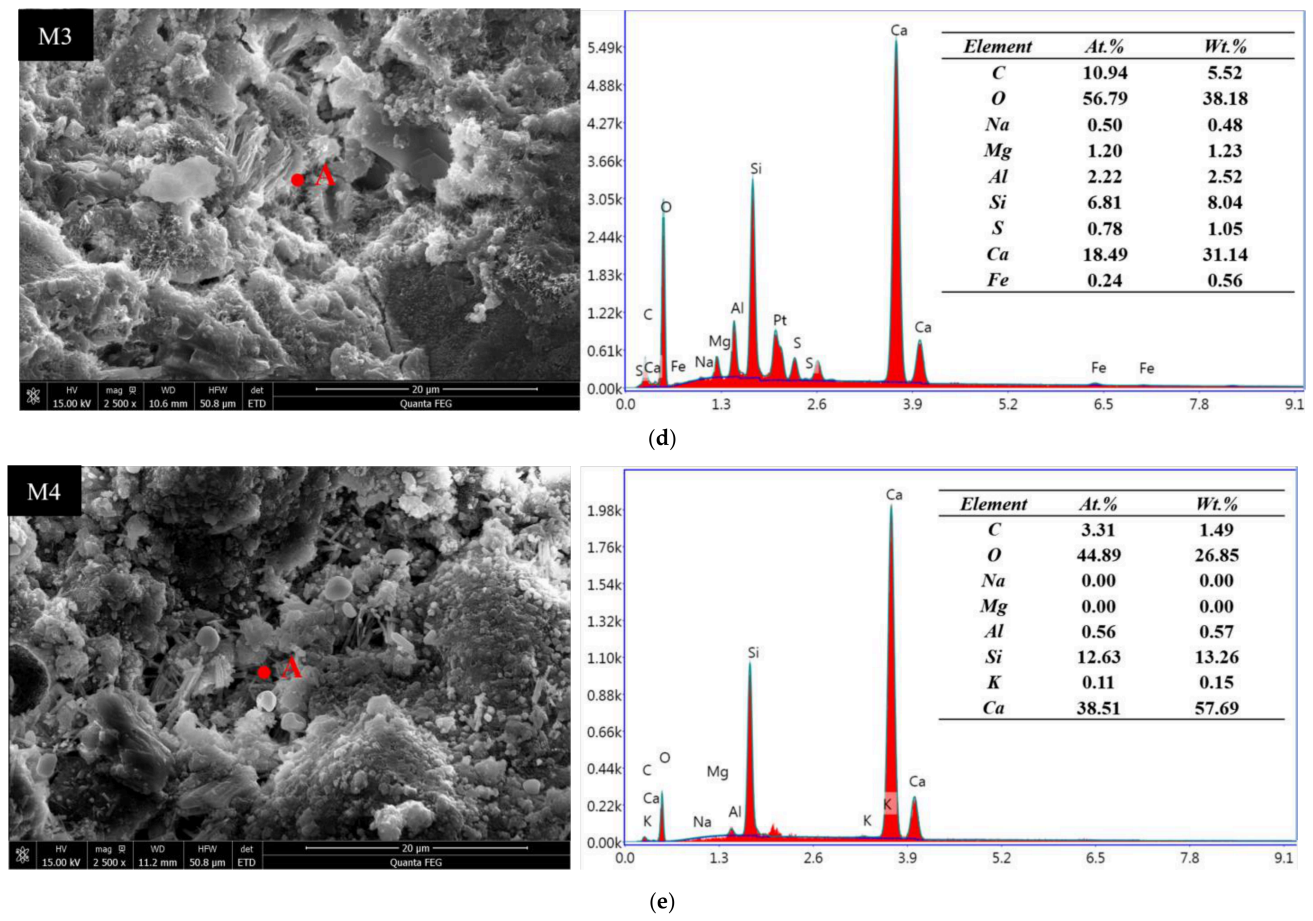
Internal microstructure of polymer-modified repair mortars: (**a**) M0; (**b**) M1; (**c**) M2; (**d**) M3; (**e**) M4.

Figure 12 shows the internal microstructure of repair mortars with varying EVA contents. As the EVA content increased, the internal structure of the paste gradually transformed from loose to dense, showing a trend consistent with the previously observed mechanical properties, water–air strength ratio, and porosity results. The structure of M0 was relatively loose, with visible pores and localized cracks, uneven distribution of hydration products, and insufficient particle bonding. Energy-dispersive spectroscopy (EDS) results indicated a high Si content and relatively low Ca content, suggesting inadequate cement paste coverage around aggregates and limited overall hydration. This structure easily forms defect pathways, resulting in a lower water–air strength ratio and interfacial bonding performance. With the addition of PAM, M1 showed improved microstructure, with tighter particle connections, reduced porosity, and local formation of flocculent and plate-like hydration products. Meanwhile, the Ca content in EDS increased significantly, indicating that PAM improved paste dispersion and promoted adequate contact between cement particles and water, enhancing hydration and structural continuity. Further addition of EVA led to M2 and M3 showing increasingly dense internal structures, with large amounts of intertwined hydration products forming a continuous network. SEM images revealed needle-like, flocculent, and plate-like hydration products, where the needle-like crystals were likely ettringite, and some blocky crystals may have been related to CaCO_3_ deposition. EDS results showed increased amounts of Ca and Al, indicating enhanced hydration and paste densification. These elemental characteristics are consistent with the SEM observations, confirming a denser distribution of hydration products and a more continuous microstructure, which contributed to the improved mechanical properties and underwater bonding performance of the EVA-modified mortars. Among these, M3 exhibited the most continuous structure, significantly reduced porosity, and a more uniform distribution of hydration products, with the appearance of polymer films. This suggests that an optimal EVA content can enhance internal structural continuity via film formation, allowing hydration products to uniformly fill pores and interfaces, corresponding to the lowest porosity, highest water–air strength ratio, and maximum interfacial flexural bonding strength. When EVA content was further increased to M4, localized particle aggregation and Ca enrichment were observed, resulting in decreased structural uniformity. Excessive EVA may lead to polymer accumulation in local regions, forming thicker polymer phases that hinder uniform hydration product distribution. Although the overall structure remained relatively dense, local heterogeneity partially limited further improvement in mechanical and interfacial properties, consistent with the trends observed in porosity and strength measurements.

## 4. Conclusions

This study systematically investigated the effects of varying EVA content on the workability, mechanical properties, underwater stability, interfacial bonding, and microstructure of PAM–EVA composite-modified underwater repair mortars. The main conclusions are as follows:

(1) The repair mortar setting time was significantly prolonged by EVA. With 8% EVA, the initial setting time of the repair mortar increased from 35 min to 47 min, and the final setting time increased from 55 min to 72 min. This moderate delay in early hydration improved workability while preventing rapid stiffening of the mortar.
(2) The mechanical properties of the repair mortar were markedly enhanced by appropriate EVA content. With 8% EVA, the 28 d flexural strength of the repair mortar increased from 7.5 MPa to 9.2 MPa, and the compressive strength increased from 44.4 MPa to 50.6 MPa. Early strength developed rapidly, with slower increments after 28 d, indicating that EVA optimized cement hydration product formation and the hardening structure of the repair mortar.
(3) The underwater hardening and interfacial bonding performance of the repair mortar were improved by EVA. With 8% EVA, the underwater flexural bonding strength of the repair mortar was 3.2 MPa at 1 d and increased to 6.5 MPa at 28 d. Meanwhile, cement loss was significantly reduced, demonstrating that EVA enhanced interfacial adhesion and matrix continuity, promoting early underwater hardening of the repair mortar.
(4) The microstructure and pore continuity of the repair mortar were optimized by EVA. With 8% EVA, the total porosity of the repair mortar at 28 d decreased from 16.8% to 13.5%, and SEM observations revealed uniform filling of pores by hydration products forming a dense network.

## Figures and Tables

**Table 1:** Chemical composition of cement (%).

	CaO	SiO_2_	Al_2_O_3_	Fe_2_O_3_	MgO	SO_3_	LOI
SAC	55.12	20.68	6.23	3.32	1.72	2.60	1.80
P·O	60.28	22.18	5.71	3.63	2.65	2.88	1.47

**Table 2:** Mix proportions of repair mortar (by mass ratio).

	SAC	P·OC	Sand	Water	Water Reducer	PAM	EVA
M0	80	20	150	45	1.5	0	0
M1	80	20	150	45	1.5	2	0
M2	80	20	150	45	1.5	2	4
M3	80	20	150	45	1.5	2	8
M4	80	20	150	45	1.5	2	12

## Data Availability

The original contributions presented in this study are included in the article. Further inquiries can be directed to the corresponding authors.
